# Supplementary material for: Rational Design and Synthesis of HSF1-PROTACs for Anticancer Drug Development
Source: Molecules. 2022 Mar 2;27(5):1655. doi: 10.3390/molecules27051655 (PMC8912087; doi:10.3390/molecules27051655)
Supplement: Supplementary file 1 [file molecules-27-01655-s001.zip › molecules-1604855-supplementary.pdf]

Supplementary Information

Rational Design and Synthesis of HSF1-PROTACs for Anticancer Drug Development

Chiranjeev Sharma, Myeong A Choi, Yoojin Song and Young Ho Seo*,[a]

College of Pharmacy, Keimyung University, Daegu 704-701, South Korea.

* To whom correspondence should be addressed. E-mail: seoyho@kmu.ac.kr

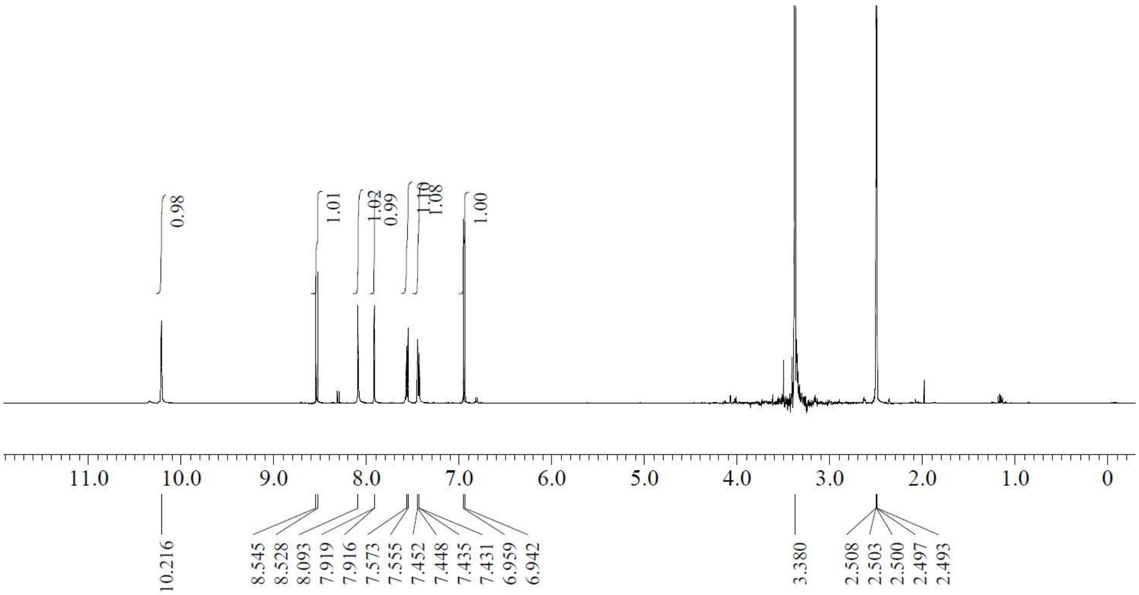

Figure S1. ^{1}H spectrum of compound **3** (500 MHz, DMSO-D_6)

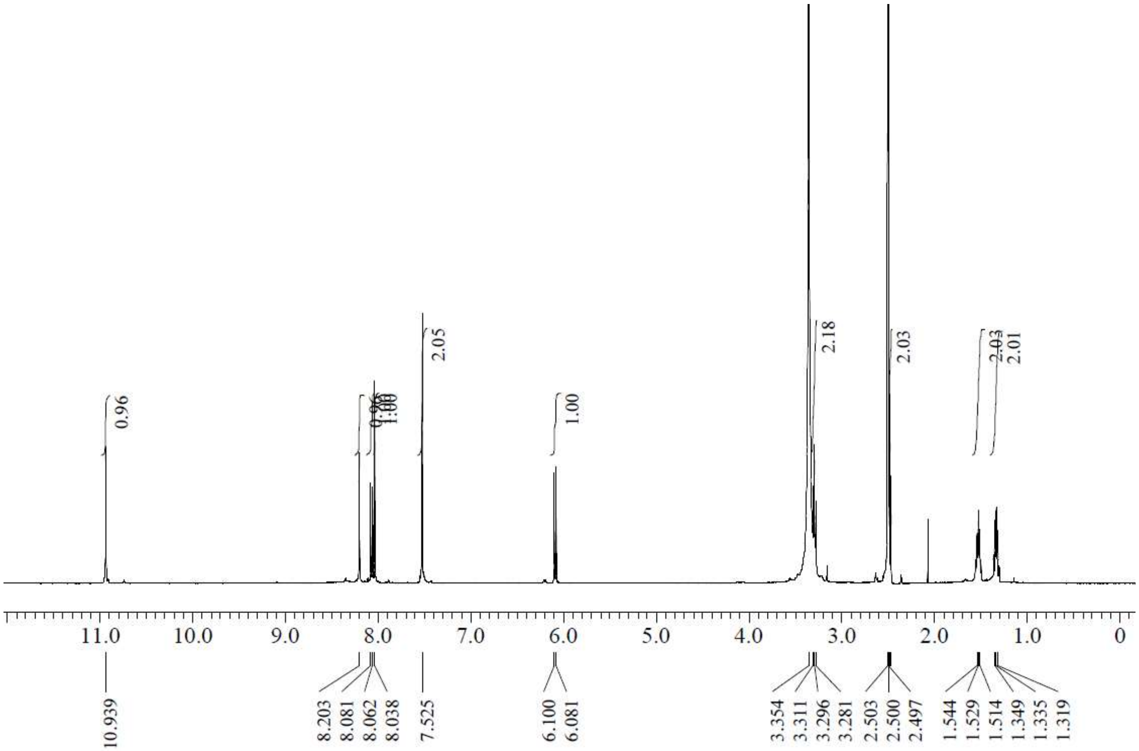

Figure S2. ^{1}H spectrum of compound **4** (500 MHz, DMSO-D_6)

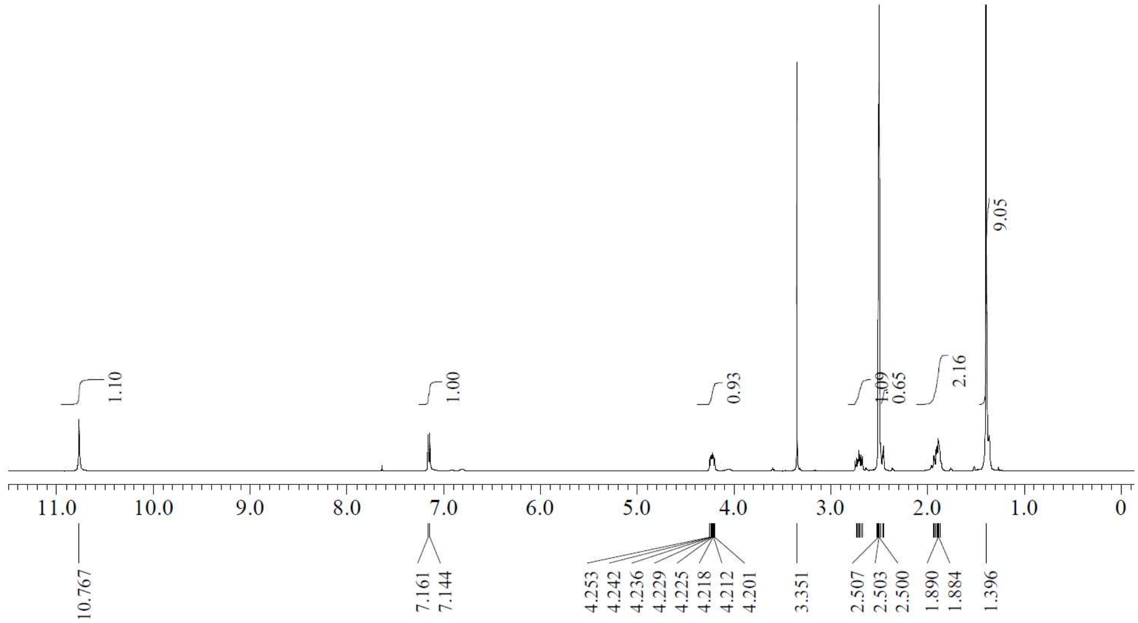

Figure S3. ^{1}H spectrum of compound **6** (500 MHz, DMSO-D_6)

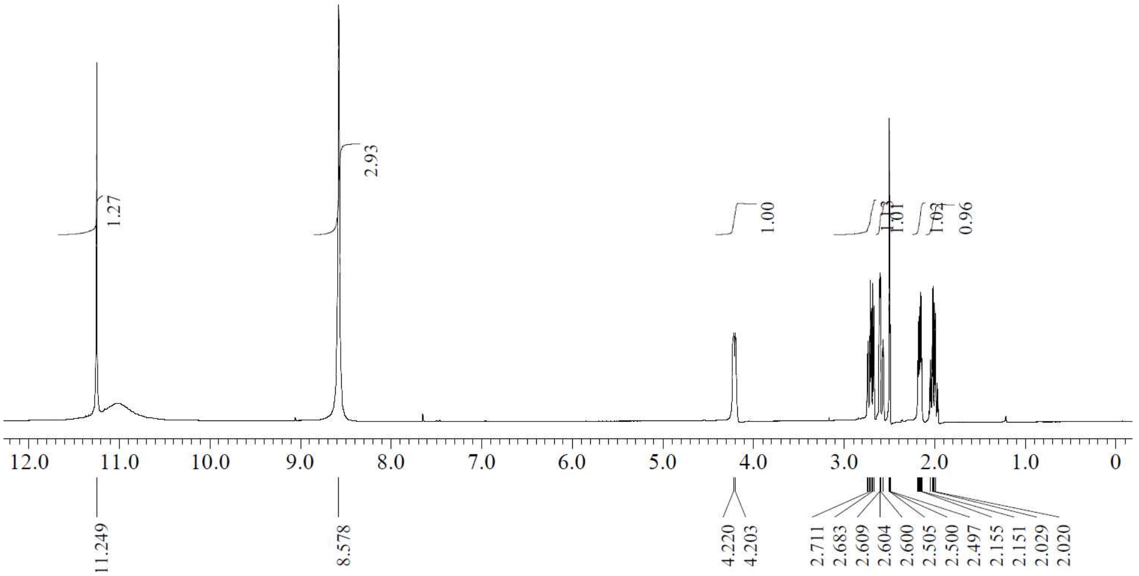

Figure S4. ^{1}H spectrum of compound **7** (500 MHz, DMSO-D_6)

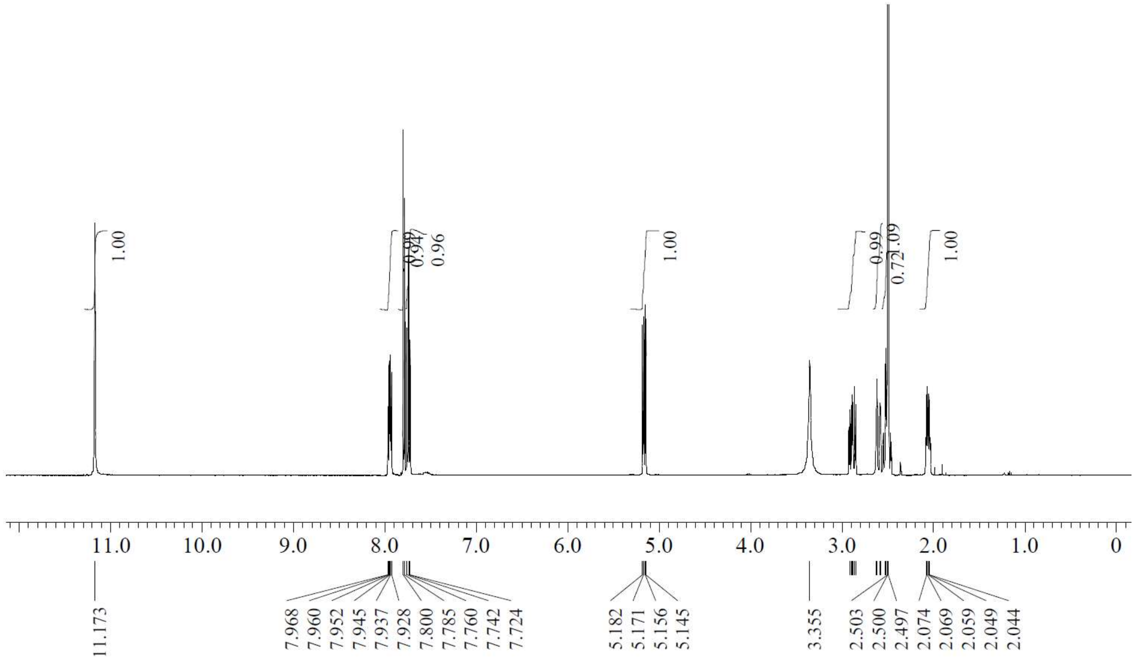

Figure S5. ^{1}H spectrum of compound **9a** (500 MHz, DMSO-D$_6$)

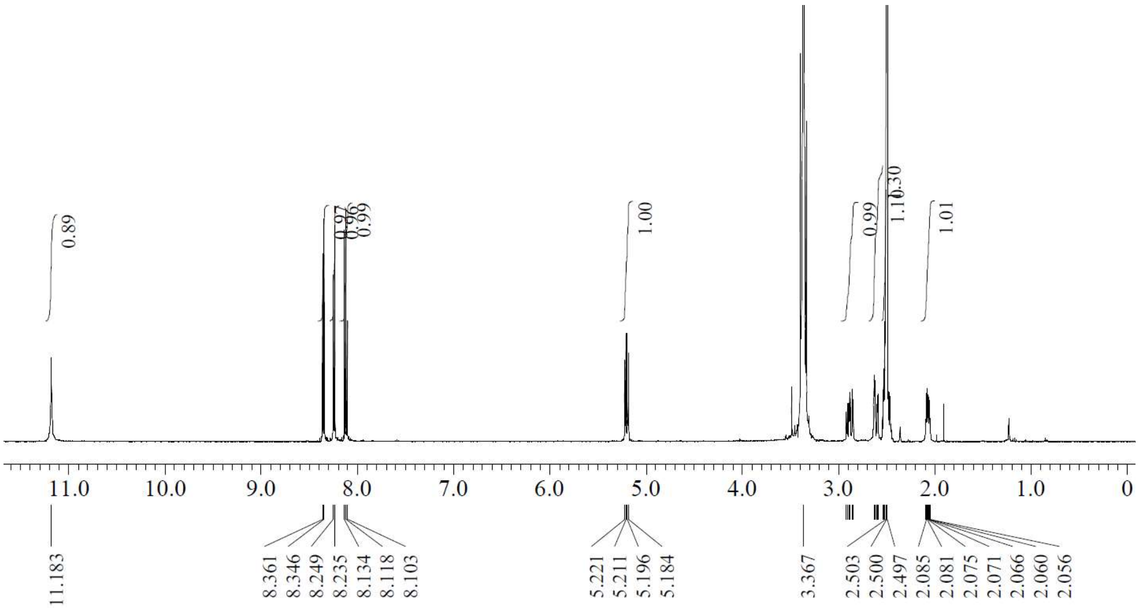

Figure S6. 1H spectrum of compound **9b** (500 MHz, DMSO-D$_6$)

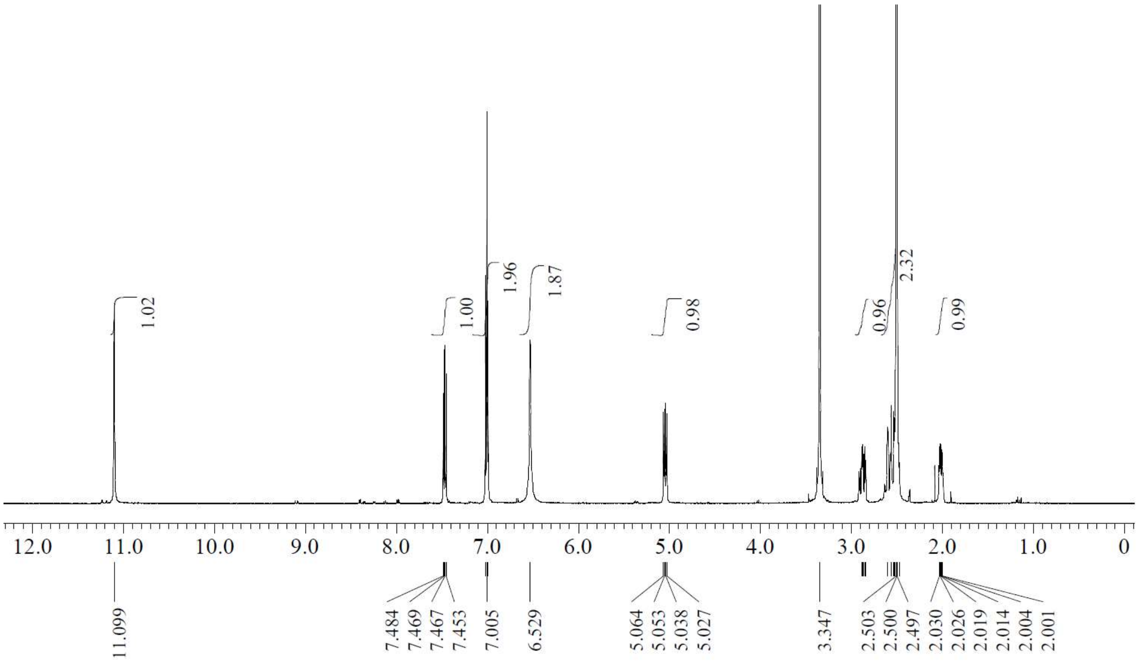

Figure S7. ^{1}H spectrum of compound **9c** (500 MHz, DMSO-D_6)

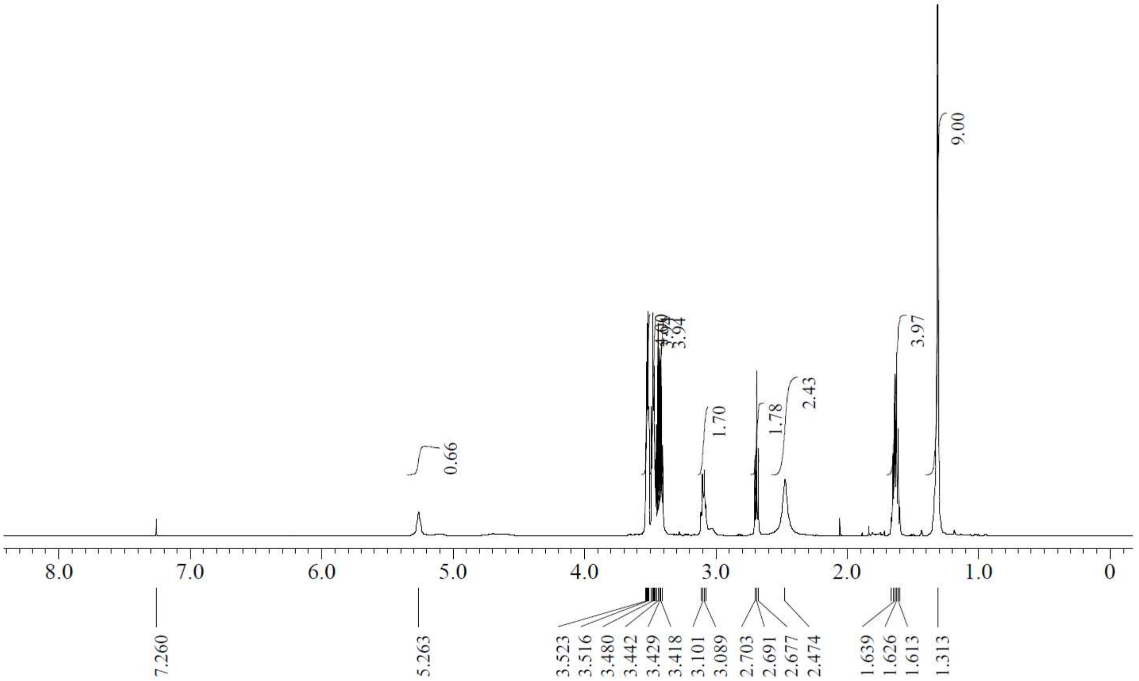

Figure S8. 1H spectrum of compound **10b** (500 MHz, $CDCl_3$)

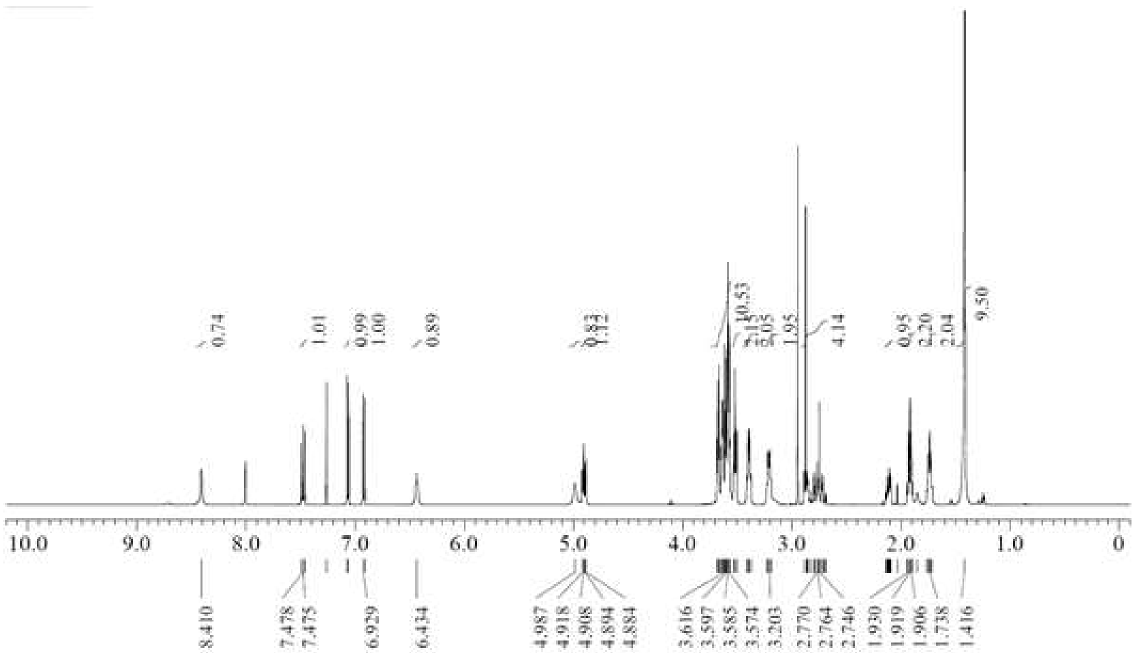

Figure S9. ^{1}H spectrum of compound **12a** (500 MHz, $CDCl_3$)

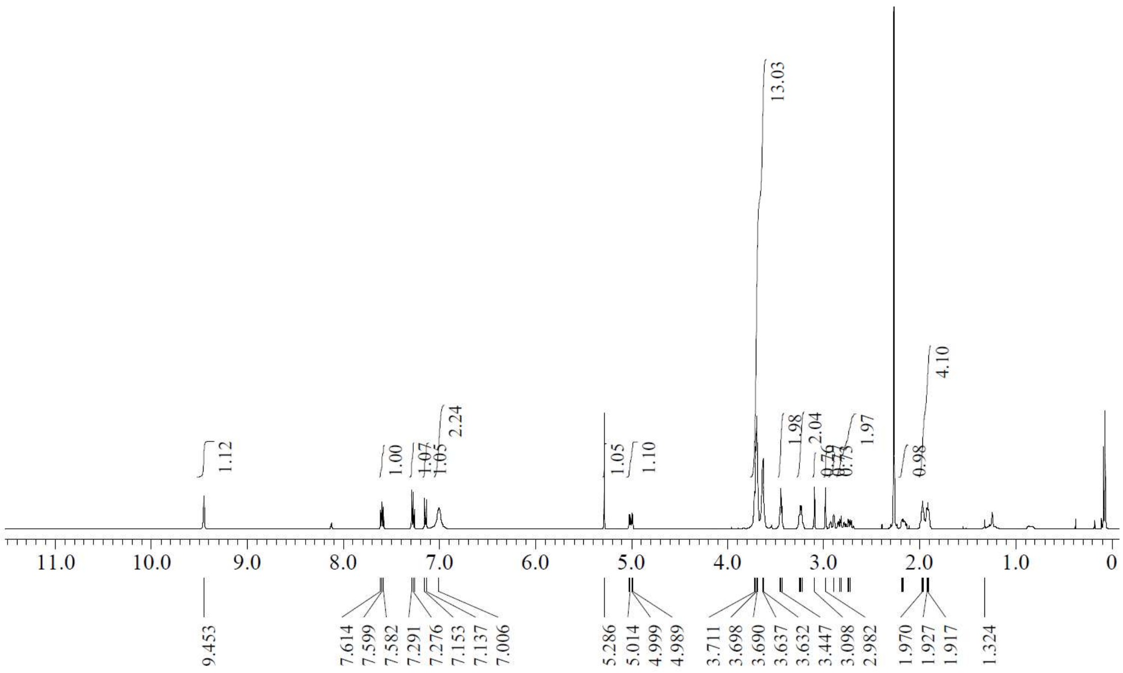

Figure S10. 1H spectrum of compound **12b** (500 MHz, $CDCl_3$)

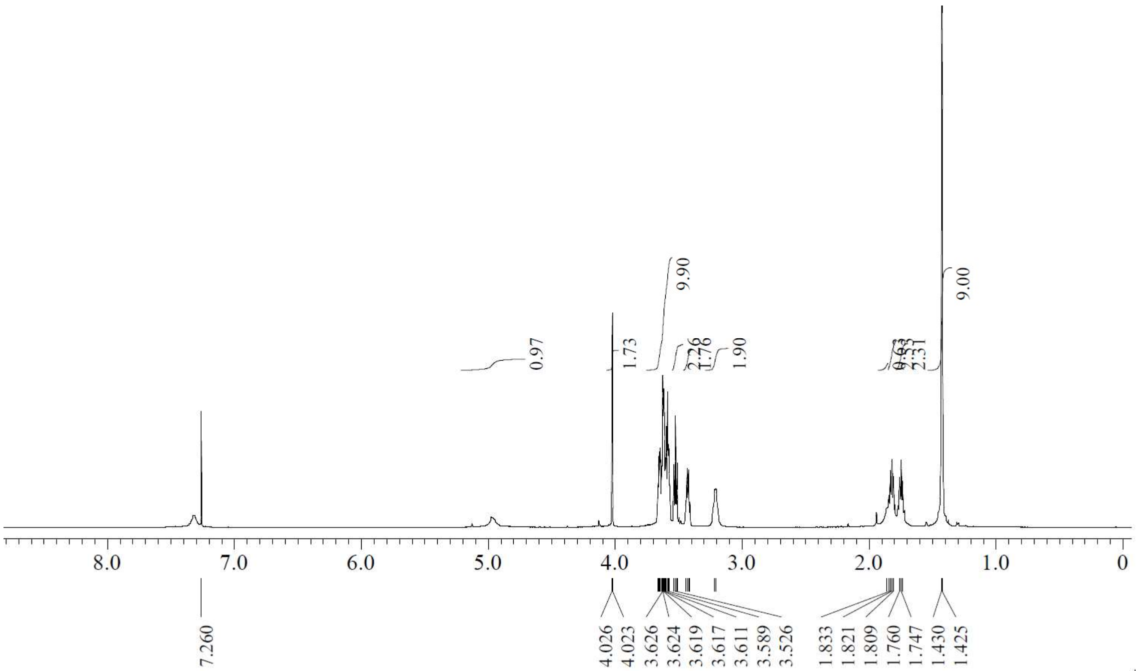

Figure S11. ^{1}H spectrum of compound **11** (500 MHz, $CDCl_3$)

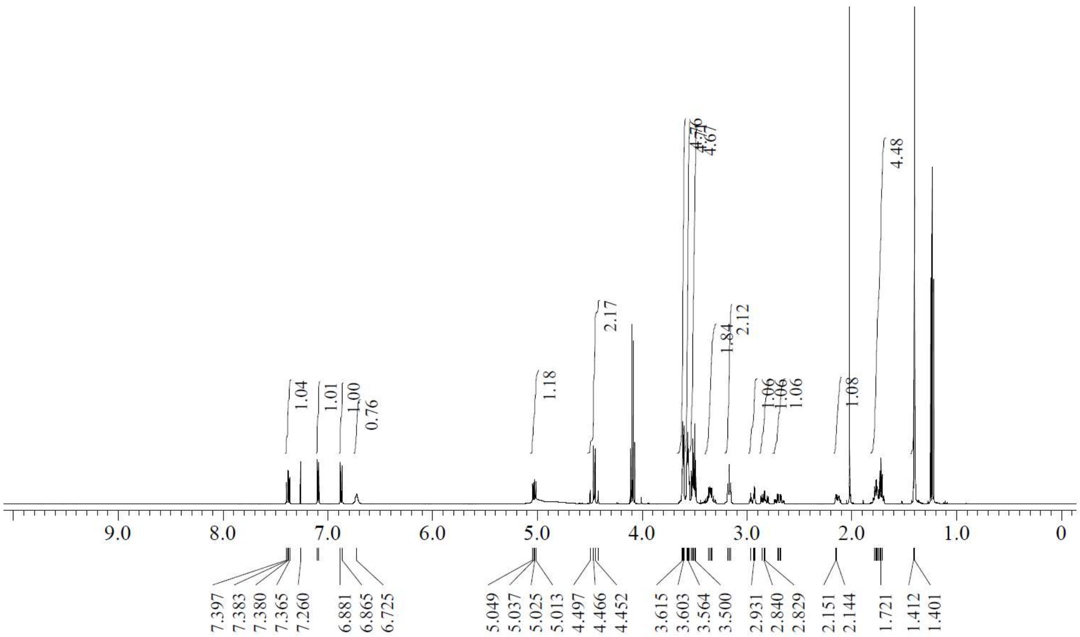

Figure S12. ^{1}H spectrum of compound **13a** (500 MHz, $CDCl_3$)

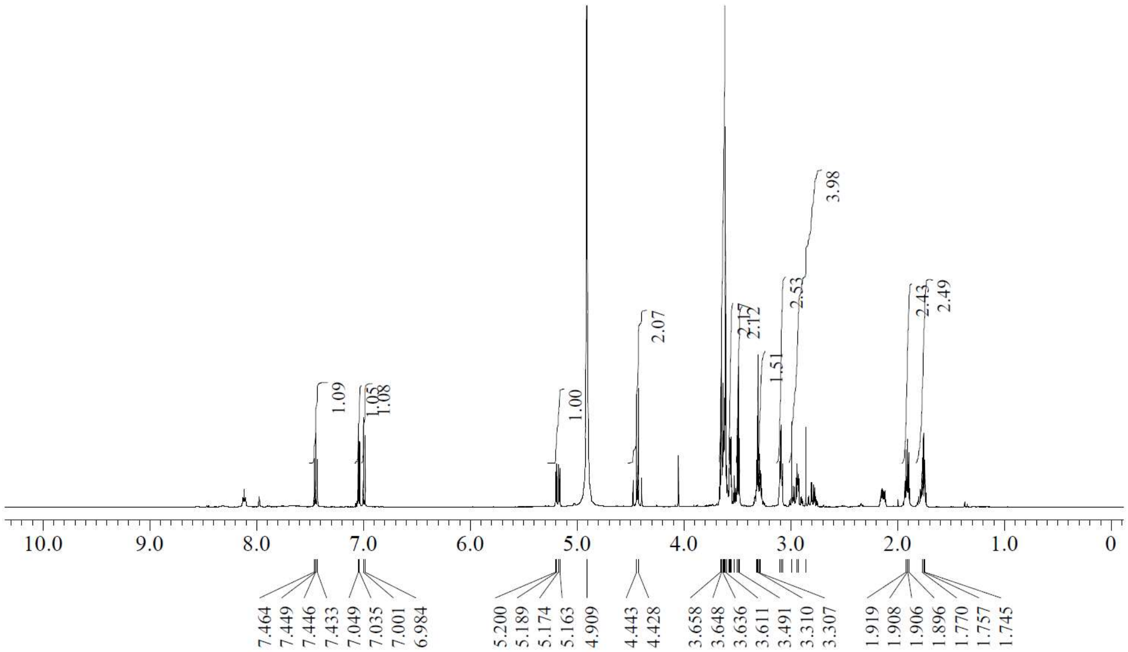

Figure S13. ^{1}H spectrum of compound **13b** (500 MHz, CD_3OD)

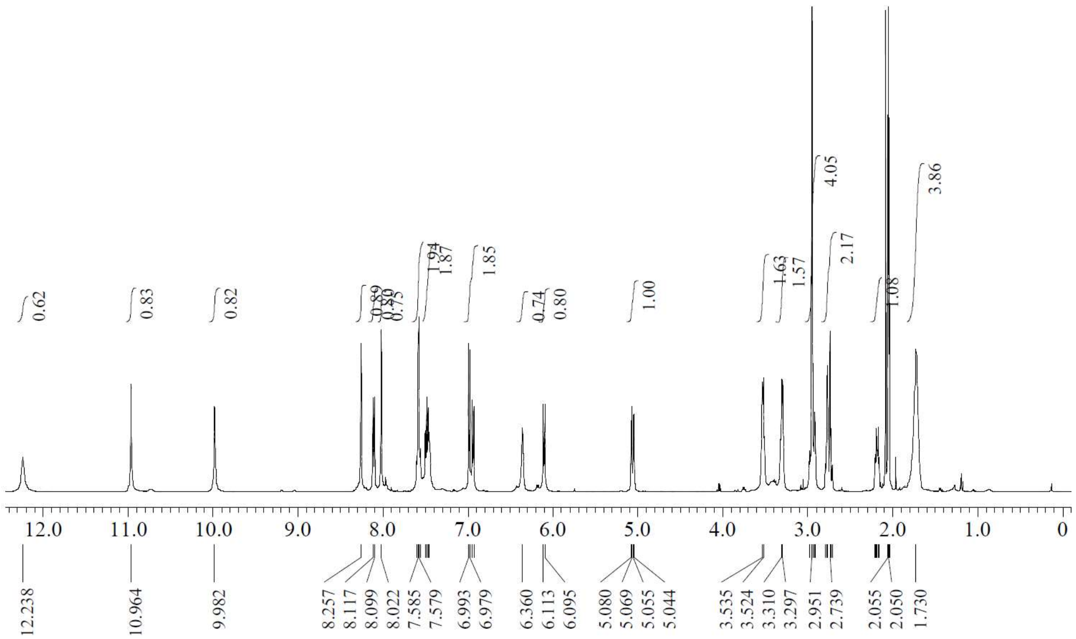

Figure S14. ^{1}H spectrum of compound **14** (500 MHz, Acetone-D_6)

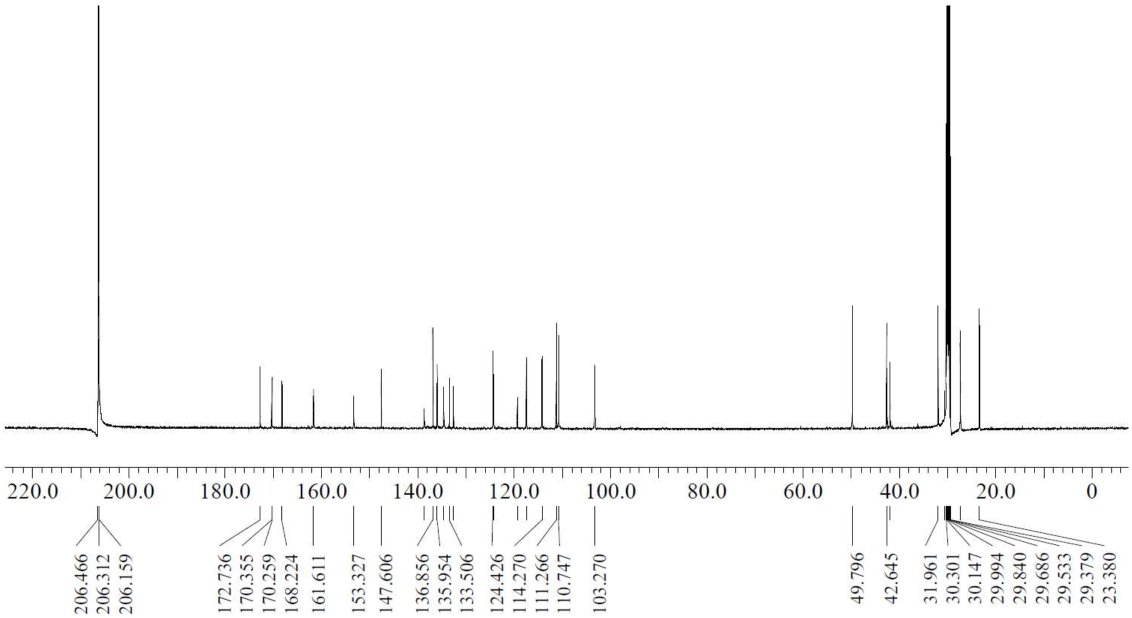

Figure S15. ^{13}C spectrum of compound **14** (126 MHz, Acetone-D_6)

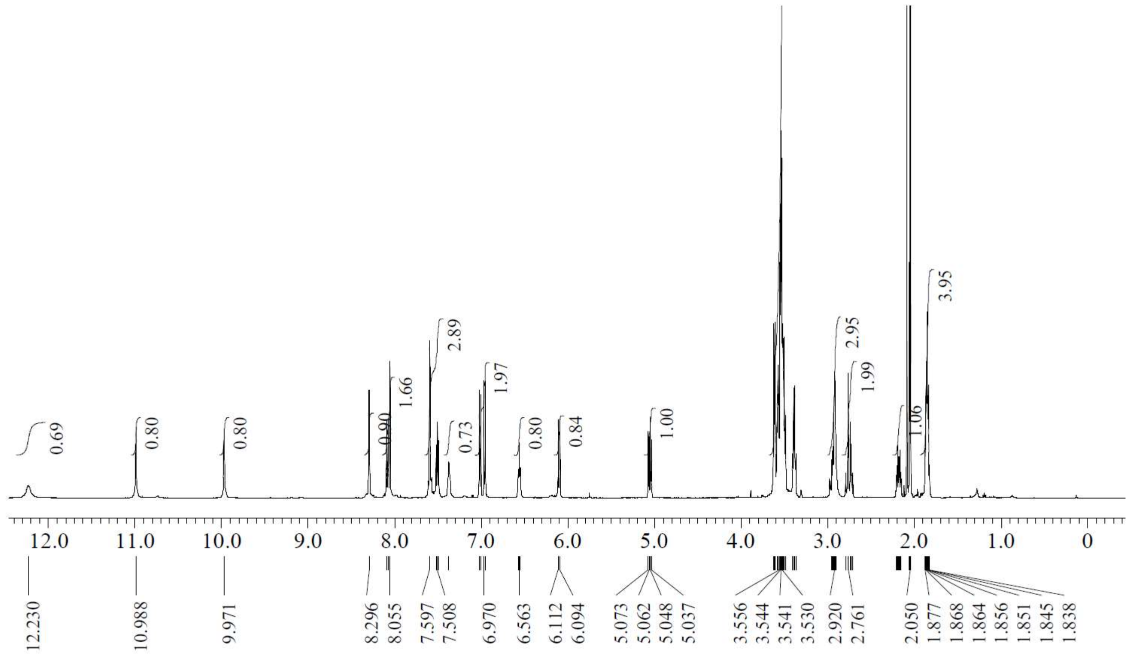

Figure S16. ^{1}H spectrum of compound **15** (500 MHz, Acetone-D_6)

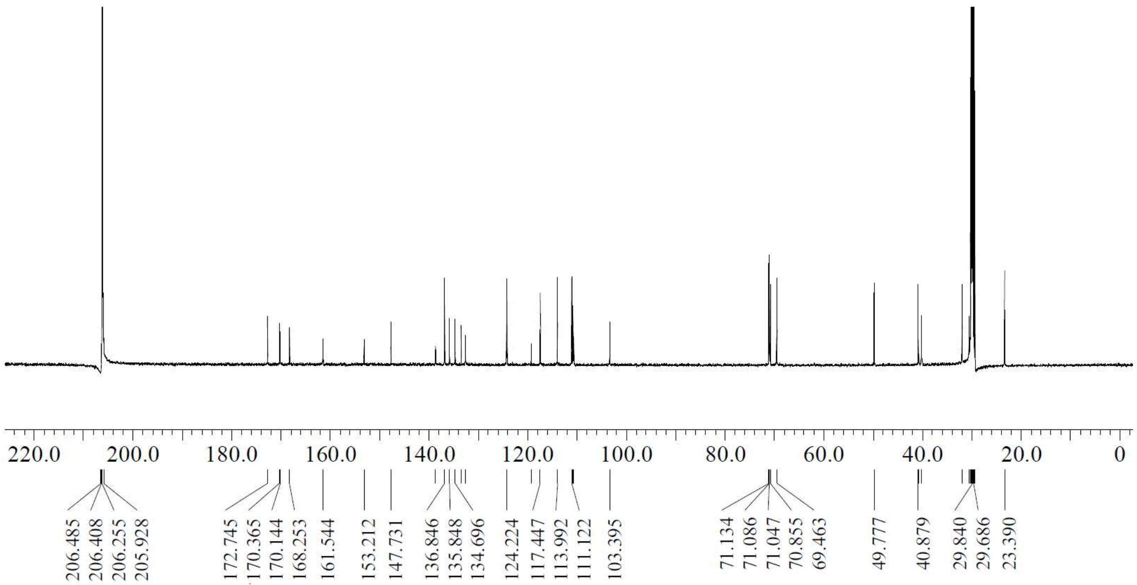

Figure S17. ^{13}C spectrum of compound **15** (126 MHz, $CDCl_3$)

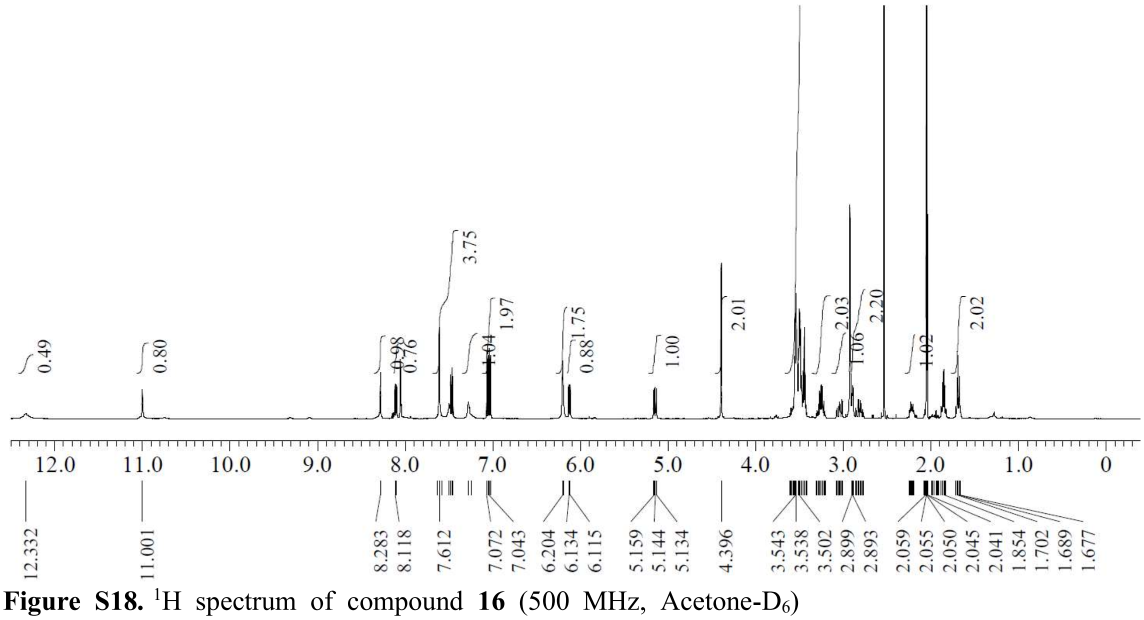

Figure S18. ^{1}H spectrum of compound **16** (500 MHz, Acetone-D_6)

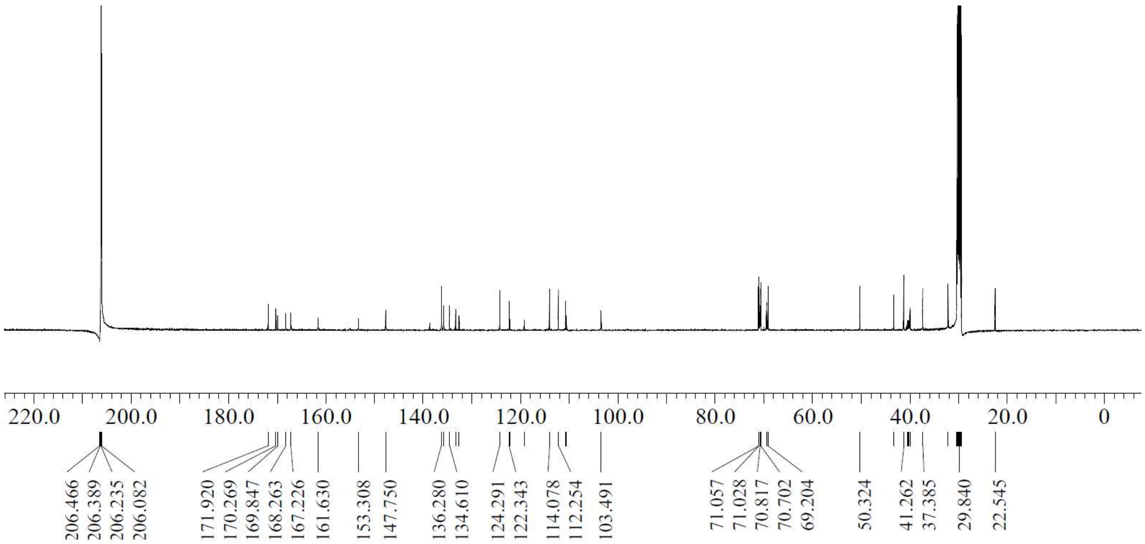

Figure S19. ^{13}C spectrum of compound **16** (126 MHz, Acetone-D_6)

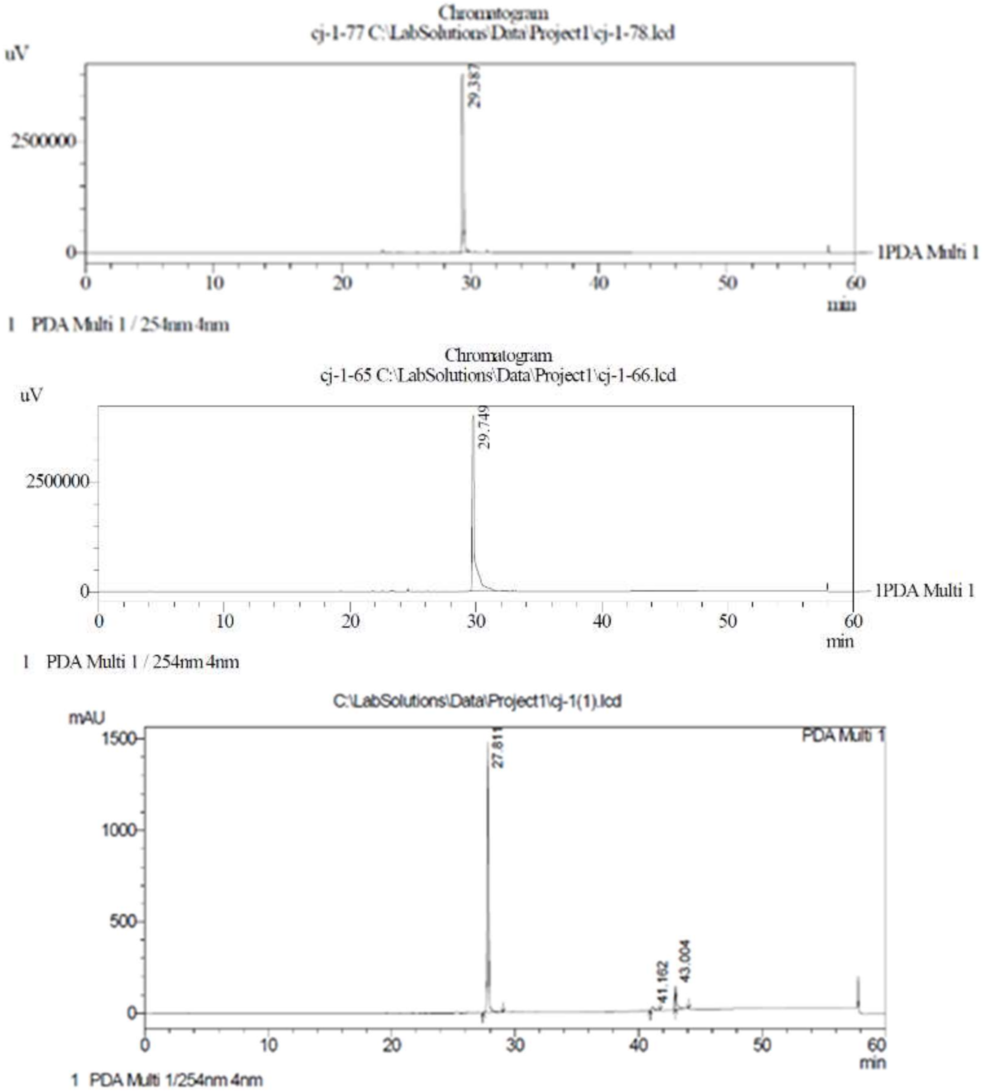

Figure S20. HPLC Chromatogram of PROTACs **14-16**.
